# Molecular Classification of Hepatocellular Carcinoma Using Wnt–Hippo Signaling Pathway-Related Genes

**DOI:** 10.3390/cancers14194580

**Published:** 2022-09-21

**Authors:** Ya-Sian Chang, Yu-Pao Chou, Chin-Chun Chung, Ya-Ting Lee, Ju-Chen Yen, Long-Bin Jeng, Jan-Gowth Chang

**Affiliations:** 1 Center for Precision Medicine, China Medical University Hospital, Taichung 404332, Taiwan; 2 Epigenome Research Center, China Medical University Hospital, Taichung 404332, Taiwan; 3 Department of Laboratory Medicine, China Medical University Hospital, Taichung 404332, Taiwan; 4 School of Medicine, China Medical University, Taichung 404333, Taiwan; 5 Organ Transplantation Center, China Medical University Hospital, Taichung 404332, Taiwan; 6 Department of Bioinformatics and Medical Engineering, Asia University, Taichung 41354, Taiwan

**Keywords:** Taiwanese hepatocellular carcinoma, molecular classification, Wnt–Hippo pathways, immune infiltration

## Abstract

**Simple Summary:**

The characters of Taiwanese hepatocellular carcinoma (HCC) are different from other parts of the world. We characterized the molecular features of HCC using a newly developed classification system based on the expression of the Wnt–Hippo signaling pathway-related genes. We analyzed the data in terms of prognostic value, transcriptome features, immune infiltration, and clinical characteristics, and compared the resulting subclasses with previously published classifications. A new molecular classification method based on a 272 gene panel of Wnt–Hippo pathways that may provide a new target for the treatment.

**Abstract:**

In Taiwan, a combination of hepatitis B and C infection, economic boom-related food and alcohol overconsumption, and Chinese medicine prescriptions has led to a high rate of hepatocellular carcinoma (HCC). However, the causative factors and underlying tumor biology for this unique HCC environment have not been identified. Wnt and Hippo signaling pathways play an important regulatory role in HCC development, and their functions are generally considered as positive and negative regulators of cell proliferation, respectively. In this study, we characterized the molecular features of HCC using a newly developed classification system based on the expression of the Wnt–Hippo signaling pathway-related genes. RNA sequencing (RNA-Seq) was performed on liver tumor tissues from 100 patients with liver cancer. RNA-Seq data for 272 previously characterized Wnt–Hippo signaling pathway-related genes were used for hierarchical clustering. We analyzed the data in terms of prognostic value, transcriptome features, immune infiltration, and clinical characteristics, and compared the resulting subclasses with previously published classifications. Four subclasses of HCC (HCCW1–4) were identified. Subclass HCCW1 displayed the highest *PCDHB4* expression. Subclass HCCW2 displayed lower Edmondson–Steiner grades (I and II) and *CTNNB1* mutation frequencies. Subclass HCCW3 was associated with a good prognosis, the highest *PCDHGB7* expression, high CD8+ naïve T cells abundance, and relatively low *TP53* mutation rates. Subclass HCCW4 was associated with a poor prognosis, the highest *PCDHB2* and *PCDHB6* expression, a relatively high abundance of Th1 cells, NKT and class-switched memory B cells, relatively low enrichment of cDC, iDC, and CD4+ memory T cells, and high Edmondson–Steiner grades (III and IV). We also identified Wnt–Hippo signaling pathway-related genes that may influence immune cell infiltration. We developed a panel of 272 Wnt–Hippo signaling pathway-related genes to classify HCC into four groups based on Taiwanese HCC and The Cancer Genome Atlas Liver Hepatocellular Carcinoma datasets. This novel molecular classification system may aid the treatment of HCC.

## 1. Introduction

Liver cancer is the fifth most common cancer and the second common cause of cancer-related death worldwide, and hepatocellular carcinoma (HCC) is its predominant form [1,2]. In addition to genetic factors, HCC development is associated with chronic viral infection, alcohol abuse, diabetes mellitus (DM), obesity, metabolic diseases, hemochromatosis, and autoimmune hepatitis [3,4]. The incidence of noninfectious HCC in developed countries is increasing due to increased rates of obesity, DM, and metabolic diseases [5,6]. These conditions induce liver injury and progressive inflammation, such that liver cells show a cycle of necrosis, regeneration, somatic mutations, and chromosomal instability [7,8,9]. Recent studies have revealed many genomic alterations of HCCs, and numerous frequently altered genes including *TP53*, *CTNNB1*, and *TERT* [10,11,12,13]. Surgery resection is the major treatment for HCC; however, many HCCs found at the unresected stage require chemotherapy or targeted therapy. Despite many potential therapeutic targets, few drugs have shown clinically promising effects, such as the multikinase inhibitors sorafenib and regorafenib, and immune checkpoint inhibitors; however, most of these drugs increased survival by only a few months, indicating the need for new drugs to treat HCC [4,6].

Nationwide programs have been initiated in Taiwan to reduce hepatitis B virus (HBV)- and hepatitis C virus (HCV)-related HCC, beginning in 1984; aggressive countermeasures against HBV and HCV infections have also been implemented by the government. These measures have resulted in a reduction in the number of HBV carriers and a decrease in the rate of HBV-related HCC among younger individuals [14,15,16]. There is no vaccine available for hepatitis C even nowadays. Chinese herbs are also prescribed for the treatment of diseases, including chronic hepatitis, under the national health system; these herbs may contain compounds that modulate aristolochic acid or liver toxins [17]. Moreover, an economic boom was associated with higher rates of DM, metabolic disorders, and obesity [14,18,19]. HCC in Taiwan is unique due to the higher prevalence of chronic hepatitis B than in the Western world. Nonalcoholic fatty liver disease, alcoholic liver disease, and chronic hepatitis C are more common in Western countries.

Aberrant Wnt signaling promotes the development and/or progression of HCC. Mutations of *CTNNB1*, a gene that codes for β-catenin in the Wnt signaling pathway, are common in HCC [20]. Aberrant activation of Hippo signaling pathway components has also been observed in HCC, especially the Yes-associated protein and transcriptional co-activator with PDZ-binding motif transcription factors [21]. Inactivation of the Hippo pathway is significantly associated with poor prognosis in human HCC [22]. Molecular classification of HCC plays an important role in the selection of therapeutic strategies and evaluation of therapeutic responses and prognoses [10,11,12,13,23,24,25,26]. For example, a recently developed metabolic network classified HCC patients into three subgroups (iHCC1–iHCC3), where the iHCC2 phenotype was associated with aberrant Wnt signaling: 75% of iHCC2 tumors found to carry *CTNNB1* mutations and displayed upregulated expression of β-catenin target genes [24]. Another study identified three HCC subtypes (S1–S3), where S1 tumors exhibited Wnt pathway activation through two mechanisms: *CTNNB1* mutation and TGF-β activation [25]. 

In this study, we applied bioinformatic tools for RNA sequencing (RNA-Seq) analysis of 100 Taiwanese HCCs, and then used several approaches to develop a survival-related molecular classification method. This is the first study to analyze the Wnt–Hippo pathways for the molecular classification of HCCs in Taiwan. Our findings improve our understanding of HCC etiology and provide a basis for the identification of new treatment targets.

## 2. Materials and Methods

### 2.1. Liver Samples

HCC was determined by pathological diagnosis. Tumor samples were collected and frozen at −80 °C after surgical resection at the Tissue Bank of the China Medical University Hospital (CMUH). The CMUH Tissue Bank was established in 2005 and accredited by Taiwan Government on 25 October 2012 and has collected more than 20,000 cancer tissues from patients with more than 20 cancer types. This study was approved by the Ethics Committee of the CMUH (CMUH 109-REC3-055), and written informed consent was obtained from all participants according to the standard procedure of the CMUH Tissue Bank.

### 2.2. RNA Extraction and RNA-Seq

RNA was extracted from tissue samples using the NucleoSpin RNA Kit (Macherey–Nagel GmBH, Duren, Germany) following the manufacturer’s instructions. The quality, quantity, and integrity of the RNA were evaluated using a NanoDrop1000 spectrophotometer and Bioanalyzer 2100 (Agilent Technologies, Santa Clara, CA, USA).

RNA-Seq was performed as described previously [27]. Briefly, samples with an RNA integrity number > 6.0 were used for RNA-Seq. An mRNA-focused, barcoded library was generated using the TruSeq strand mRNA Library Preparation Kit (Illumina, San Diego, CA, USA). The libraries were sequenced on the Nova Seq 6000 instrument (Illumina), using 2 × 151-bp paired-end sequencing flow cells following the manufacturer’s instructions.

### 2.3. RNA-Seq Data Analysis

The RNA-Seq data were analyzed as described previously [28]. Briefly, data quality control at the Q20 level was performed using the Trimmomatic tool [29], read alignment to the GRCh38 human genome was conducted using the HISAT2 alignment program [30], expression was quantified with reference to GENCODE v35 (excluding mitochondrial genes), and transcripts were normalized to transcripts per million (TPM) using the StringTie assembler [31].

### 2.4. Molecular Classification of HCC

Molecular classification of HCC was performed using transcriptomics-based analysis integrating Wnt [32] and Hippo [33] signaling pathways-related gene panels, coupled with survival analysis, and then validated using data from The Cancer Genome Atlas (TCGA). Subjects were also classified according to molecular signatures reported in other HCC studies [24,25]. For the molecular classification, hierarchical clustering was performed using Spearman rank correlation for sample distance calculation and the “average” linkage method using the Morpheus web tool (https://software.broadinstitute.org/morpheus/, accessed on 13 April 2022). Important nodes and subnetworks were predicted and explored using CytoHubba [34], a Cytoscape plugin [35].

### 2.5. Cell Enrichment Analysis

The xCell [36] was used to examine the enrichment of various immune cells in the tumors and to calculate an immune score from the TPM expression matrix.

### 2.6. Statistical Analyses

The Kaplan–Meier method was used to construct overall survival curves, and the *p*-values were obtained using a log-rank test. The Kruskal–Wallis test and Mann–Whitney U test were used to compare groups in terms of non-normally distributed variables. Contingency table variables were analyzed using Fisher’s exact test. All statistical analyses were performed in the Python programming environment. A two-tailed *p* < 0.05 was considered statistically significant.

## 3. Results 

### 3.1. Molecular Subtypes of Taiwanese HCC Based on the Expression Profiles of 254 Wnt Pathway Genes

**Figure 1:**
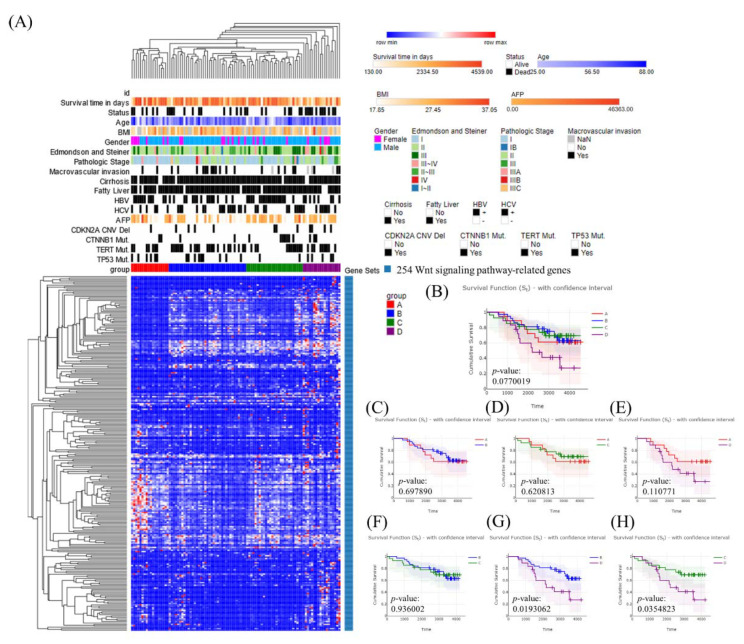
Molecular classification of Taiwanese hepatocellular carcinoma (HCC) using a panel of 254 Wnt pathway genes. (**A**) Heatmap showing normalized expression levels of 254 signature genes (rows) across subjects (columns) divided into four molecular subclasses (**A**–**D**) using an unsupervised approach. Each signature gene was significantly up- or downregulated in one subclass relative to the other subclasses, as indicated by color changes, where blue and red indicate low and high expression, respectively. Signature genes and subjects were hierarchically clustered within each subclass. Significant differences in demographic characteristics, clinical annotations, molecular subclasses, and hepatitis B and C virus infection status across subjects are shown at the top of the figure. (**B**–**H**) Overall survival of subjects in each molecular subclass.

Hierarchical clustering analysis identified four subclasses (A–D) of Taiwanese HCC patients (Figure 1A). Kaplan–Meier plots of the results of individual and grouped pairwise analysis showed significant differences in survival between subclasses B and D (*p* = 0.019) and C and D (*p* = 0.035), but there was no significant difference in a comparison of all four groups (*p* = 0.077) (Figure 1B–H). Moreover, we investigated the expression of *CTNNB1* up/downstream genes for *CTNNB1* overexpression or down expression. *FZD1*, *FZD3*, *FZD4*, *FZD5*, *FZD6*, *FZD7*, *GSK3B*, *TCF7L2*, *TLE4*, *LRP5*, *TLE2*, *TLE5*, and *TLE6* were found to be differentially expressed in *CTNNB1* overexpression or down expression (Supplementary Figure S1). We found positive correlation for the expression of *CTNNB1* with the expression of *FZD1*, *FZD3*, *FZD4*, *FZD5*, *FZD6*, *FZD7*, *GSK3B*, *TCF7L2*, and *TLE4*. However, the expression of *CTNNB1* was inversely correlated with the expression of *LRP5*, *TLE2*, *TLE5*, and *TLE6*.

### 3.2. Molecular Subtypes of Taiwanese HCC Based on the Expression Profiles of 18 Hippo Pathway Genes

**Figure 2:**
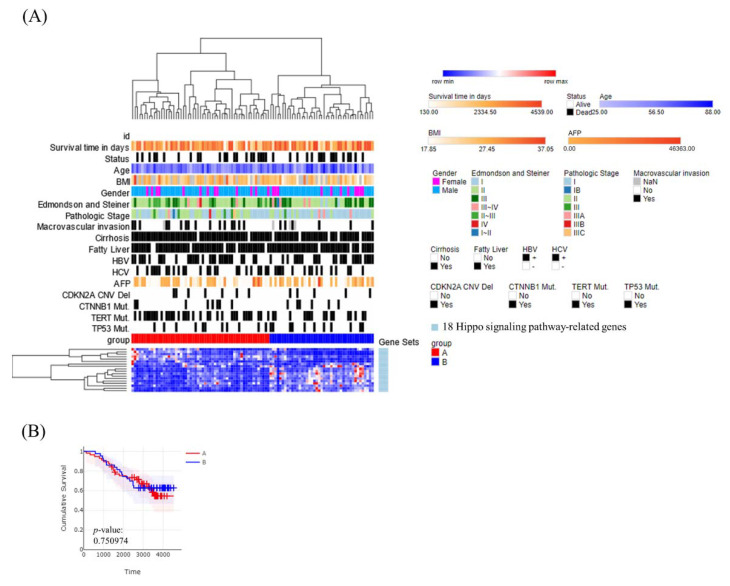
Molecular classification of Taiwanese HCC patients using a panel of 18 Hippo pathway genes. (**A**) Heatmap showing normalized expression levels of the 18 signature genes (rows) across subjects (columns) divided into two molecular subclasses (**A**,**B**) using an unsupervised approach. Each signature gene was significantly up- or downregulated compared with the other subclasses, as indicated by color changes, where blue and red indicates low and high expression, respectively. Signature genes and subjects were hierarchically clustered within each class. Significant differences in demographic characteristics, clinical annotations, molecular subclasses, and hepatitis B and C virus infection status across subjects are shown at the top of the figure. (**B**) Overall survival of subjects in each molecular subclass.

Hierarchical clustering analysis identified two subclasses (A and B) of Taiwanese HCC patients (Figure 2A). A Kaplan–Meier plot of overall survival showed no significant difference between the two subclasses (*p* = 0.751) (Figure 2B). In addition, we examined the expression of *YAP1* up/downstream genes for *YAP1* overexpression or down expression. We found *TAOK1*, *TAOK2*, *TAOK3*, *SAV1*, *LATS1*, *LATS2*, *STK4*, *MOB1A*, *MOB1B*, *NF2*, *TAZ*, *TEAD1*, *TEAD3*, *TEAD4*, and *FRMD6* were differentially expressed in *YAP1* overexpression and down expression (Supplementary Figure S2) and positive correlation for the expression of *YAP1* with the expression of *TAOK1*, *TAOK3*, *SAV1*, *LATS1*, *LATS2*, *STK4*, *MOB1A*, *MOB1B*, *TEAD1*, and *FRMD6*. However, the expression of *YAP1* was inversely correlated with the expression of *TAOK2*, *NF2*, *TAZ*, *TEAD3*, and *TEAD4*.

### 3.3. Molecular Subtypes of Taiwanese HCC Based on the Expression Profiles of 272 Wnt–Hippo Pathways Genes

**Figure 3:**
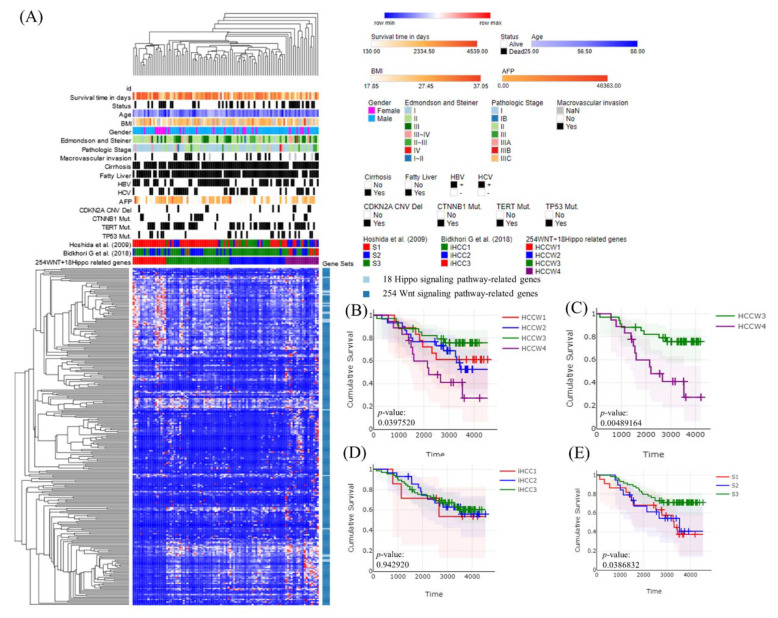
Molecular classification of Taiwanese HCC patients using a panel of 272 Wnt–Hippo pathway genes. (**A**) Heatmap showing normalized expression levels of the 272 signature genes (rows) across subjects (columns) divided into four molecular subclasses (HCCW1–4), where blue and red indicate low and high expression, respectively. Significant differences in demographic characteristics, clinical annotations, molecular subclasses, and hepatitis B and C virus infection status across subjects are shown at the top of the figure. (**B**) Overall survival for subjects in each molecular subclass. (**C**) HCCW3 showed significantly better prognosis than HCCW4 based on two-sided log-rank tests. (**D**) The Bidkhori classification results were not significantly correlated with survival, whereas (**E**) the Hoshida classification results did show a significant correlation.

A panel of Wnt pathway-related genes showed that some were corrected with survival; therefore, we added several other functional gene panels to improve the power of the analysis. Finally, among 18 Hippo pathway- and 254 Wnt pathway-related genes, we identified four molecular subclasses (HCCW1-4) (Figure 3A) that were correlated with patient’s survival (*p* = 0.040) (Figure 3B). The average survival time was significantly shorter for HCCW4 (2575 days; 95% confidence interval (CI): 1953–3197, n = 18) than for HCCW2 (3075 days; 95% CI: 2649–3502, n = 30), HCCW1 (3446 days; 95% CI: 2792–4101, n = 18), and HCCW3 (3556 days; 95% CI: 3143–3968, n = 34). HCCW3 was associated with a statistically significantly better prognosis than HCCW4 based on a two-sided log-rank test (*p* = 0.005) (Figure 3C). 

**Figure 4:**
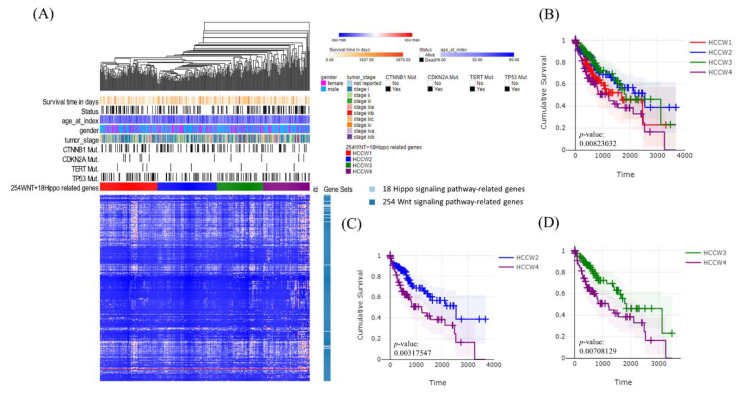
Molecular classification of HCC data from The Cancer Genome Atlas (TCGA) using a panel of 272 Wnt–Hippo pathway genes. (**A**) Heatmap showing normalized expression levels of 272 signature genes (rows) across subjects (columns) divided into four molecular subclasses (HCCW1–4), where blue and red indicate low and high expression, respectively. Significant differences in demographic characteristics, clinical annotations, molecular subclasses, and hepatitis B and C virus infection status across subjects are shown at the top of the figure. (**B**) Overall survival for subjects in each molecular subclass. (**C**) HCCW2 showed significantly better prognosis than HCCW4 based on two-sided log-rank tests. (**D**) HCCW3 showed significantly better prognosis than HCCW4 based on two-sided log-rank tests.

To validate the findings, we applied a similar molecular classification approach using HCC (LIHC) data from TCGA and obtained similar results (*p* = 0.008; Figure 4A,B). HCCW4 showed significantly lower survival than HCCW2 (*p* = 0.003) (Figure 4C) and HCCW3 (*p* = 0.007) (Figure 4D), with a more distinct grouping than that seen for Taiwanese HCC patients, perhaps due to a larger sample size. The Bidkhori classification method [24] applied to our cases based on a panel of 51 metabolic genes revealed no correlation with survival (*p* = 0.943) (Figure 3D), whereas the results of the Hoshida classification [25] showed a correlation with survival (*p* = 0.039) (Figure 3E). 

### 3.4. Transcriptomes of the HCC Subclasses

**Figure 5:**
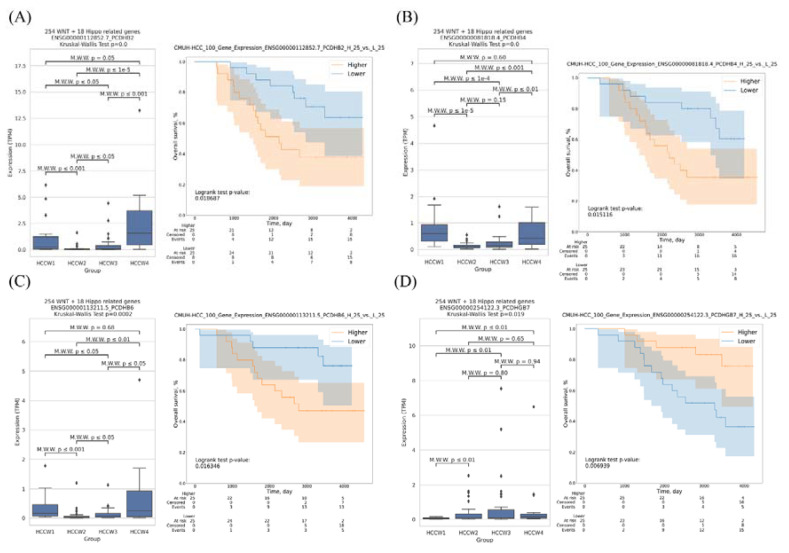
Correlations between molecular classifications and patient survival. Boxplot showing the expression level of (**A**) *PCDHB2*, (**B**) *PCDHB4*, (**C**) *PCDHB6*, and (**D**) *PCDHGB7* in four HCC subclasses. The statistical difference was compared through the Kruskal–Wallis test and Mann–Whitney U test, and the *p*-values were shown above each boxplot. Survival analysis of patients with HCC according to above-mentioned genes expression. Patients with high *PCDHB2*, *PCDHB4*, and *PCDHB6* expression had significantly shorter overall survival compared with patients with low expression.

We further analyzed the expression of genes that may influence the molecular classification of Taiwanese HCC patients, the results showed differential expression of many genes among the four groups; however, we detected no correlations with survival (Supplementary Table S1). We further analyzed survival-related expression genes and found that *PCDHB6*, *PCDHGB7*, *PCDHB2*, and *PCDHB4* were correlated with the molecular classification and patient survival (Figure 5A–D and Supplementary Table S1). Patients with high *PCDHB6*, *PCDHB2*, and *PCDHB4* mRNA expression levels had lower overall survival. By contrast, high *PCDHGB7* mRNA expression was associated with longer survival. We used similar approaches to analyze the TCGA data, and the results showed that 56 genes were correlated with both the molecular classification and survival (Supplementary Table S1).

**Figure 6:**
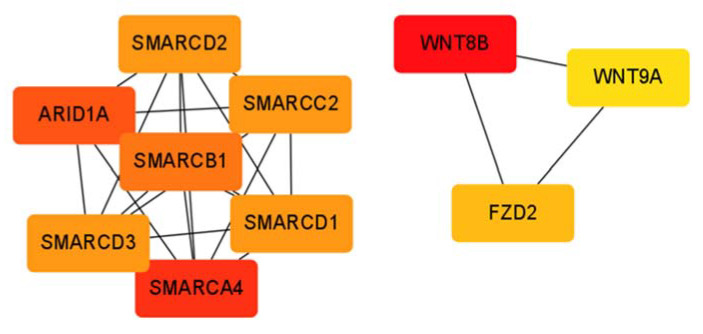
Ten upregulated hub genes in the HCCW4 subclass. The shade of the color indicates the degree of importance of the genes. The sequence of colors is red–orange–yellow from most importance to less importance.

Among the significantly differentially expressed genes related to the Wnt–Hippo pathway, 110 and 14 were up- and downregulated, respectively, in HCCW4 compared with those in HCCW1–3 (Supplementary Table S2). The most important nodes and subnetworks of protein–protein interactions among the 110 upregulated genes in HCCW4 were predicted and explored using CytoHubba; the 10 most significant node genes were *WNT8B*, *SMARCA4*, *ARID1A*, *SMARCB1*, *SMARCC2*, *SMARCD1*, *SMARCD2*, *SMARCD3*, *FZD2*, and *WNT9A* (Figure 6).

We also identified 26 and 11 differentially expressed genes that were up- and downregulated, respectively, in HCCW3 compared with those in HCCW1, HCCW2, and HCCW4 (Supplementary Table S3).

### 3.5. Correlation of HCC Subclasses with Immune Infiltration

**Figure 7:**
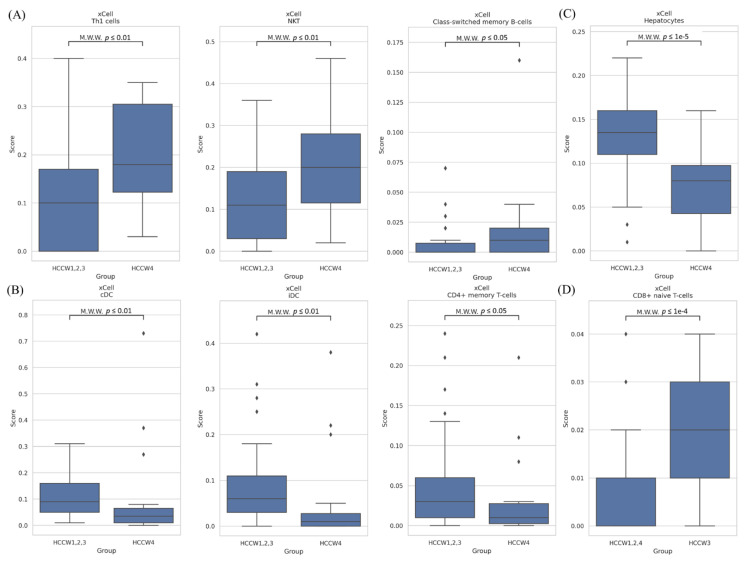
Immune characteristics of HCC subclasses. (**A**,**B**) Boxplots of immune cells differing between the subgroup with the worst prognosis (HCCW4) and the other subgroups. (**C**) Boxplot of hepatocytes differing between the subgroup with the worst prognosis (HCCW4) and the other subgroups. (**D**) Boxplots of immune cells differing between the subgroup with the best prognosis (HCCW3) and the other subgroups. The statistical difference was compared through the Mann–Whitney U test, and the *p*-values were shown above each boxplot.

We investigated the associations between the subclasses and the expression levels of 34 immune cells. Significant differences were observed between HCCW4 and the other three subclasses, with higher abundance seen for three immune cell populations (Th1, natural killer T (NKT), and class-switched memory B-cells) in HCCW4 than compared with HCCW1-3 (Figure 7A), and lower enrichment for three immune cell populations (cDC, iDC, and CD4+ memory T-cells) (Figure 7B). In the nonhematopoietic group, hepatocytes were significantly less enriched in HCCW4 (Figure 7C). In addition, CD8+ naïve T-cells were more abundant in HCCW3 (Figure 7D). 

### 3.6. Correlation of the HCC Subclasses with Clinical Characteristics 

Next, we explored the tumor-related clinicopathological variables associated with our classification results. Fisher’s exact test revealed significant correlations between clinicopathological features and HCC subclasses in our cohort. A lack of advanced Edmondson–Steiner grade (*p* = 0.0007) and lower *CTNNB1* mutation frequency (*p* = 0.0586) were associated with the HCCW2 subclass, and Edmondson–Steiner grade III/IV (*p* = 0.0203) was associated with HCCW4 subclass. HCCW3 was negatively correlated with the *TP53* mutation rate (*p* = 0.0049) (Table 1).

We also compared our classification results with those of the Bidkhor (iHCC1-3) and Hoshida (S1–S3) classification systems. HCCW3 was marginally significantly associated with the Hoshida S3 class (*p* = 0.0526), whereas HCCW4 was significantly associated with the Bidkhor iHCC3 class (*p* < 0.0001) and Hoshida S1 and S2 classes (*p* = 0.0039) (Table 1).

## 4. Discussion

Comprehensive integrated analysis of HCC is a powerful tool for understanding molecular events relevant to HCC [10,11,12,13,24,25]. In this study, we used a functional gene panel approach to explore correlations of molecular events with molecular changes and survival and devised a new molecular panel including 254 and 18 Wnt and Hippo signaling pathway-related genes, respectively, to classify Taiwanese HCC patients into four molecular subgroups (HCCW1-4). Then, the prognostic value, transcriptome features, immune infiltration, and clinical characteristics of the subgroups were explored. 

The expression of *PCDHB4* was highest for HCCW1. HCCW2 was associated with relatively low Edmondson–Steiner grade (I–II) and a relatively low *CTNNB1* mutation frequency rate. HCCW3 was associated with a good prognosis, the highest expression of *PCDHGB7*, relatively high CD8+ naïve T-cell abundance, and relatively lower *TP53* mutation rates. HCCW4 was associated with a poor prognosis, the highest *PCDHB2* and *PCDHB6* expression levels, relatively high abundance of four immune cell populations (Th1, NKT, and class-switched memory B-cells), relatively low enrichment of seven immune cell populations (cDC, iDC, and CD4+ memory T-cells), and relatively high Edmondson–Steiner grade (III–IV). Our method was also applied to TCGA HCC data; the results showed that the subgroup with the poorest survival (HCCW4) was well correlated with Hoshida classification groups S1 and S2, and Bidkhori metabolic classification group iHCC3 [24,25]. 

PCDHs are a group of cell adhesion molecules in the cadherin superfamily that can be divided into clustered (cPCDHs) and nonclustered molecules (ncPCDHs). PCDHs can interact with intracellular molecules including components of the WAVE complex, Wnt pathway, and apoptotic cascades. PCDHs are expressed prominently in the central nervous system, where they play important neurobiological roles. They are also involved in cancer development; loss or dysregulation of PCDHs is associated with multiple types of cancer [37]. Most *PCDH* genes are considered to be tumor suppressors, and epigenetic mechanisms govern their expression through their unique genomic organization that allows for long-range epigenetic silencing [37,38]. Several studies have shown that *PCDH* genes are also involved in liver cancer development, including *PCDH10*, *PCDH17*, *PCDH19*, *PCDH20*, *PCDHGC4*, and *PCDHGC5* [39,40,41,42,43]. Alterations of these *PCDHs* can occur through several oncogenic pathways; for example, *PCDH20* functions as a tumor-suppressor gene by antagonizing the Wnt/β-catenin signaling pathway in HCC [43]. In this study, *PCDHB2*, *PCDHB4*, and *PCDHB6* were overexpressed in the HCCW4 subgroup, in association with worse survival; this finding contradicts a tumor suppressor role. Our *PCDHB6* results were confirmed by the TCGA-LIHC data. Aberrant expression of these genes may have resulted from context-dependent differences among *PCDHs*. We also found that *PCDHGB7* upregulation was correlated with better HCC survival, which suggests a tumor suppressor role for *PCDHGB7* in HCC. Moreover, we examined the *PCDHB2*, *PCDHB4*, *PCDHB6*, and *PCDHGB7* mRNA and protein expression level by TCGA and Human Protein Atlas [44] databases, respectively. Among them, the RNA expression of *PCDHB4* and *PCDHGB7* were correlated with the protein level in LIHC. However, *PCDHB2* and *PCDHB6* expression were detected only at the mRNA level. *PCDHB2* and *PCDHB6* protein levels were not observed in >50% of liver cancer specimens by immunohistochemistry.

Wnt signaling pathway components play important regulatory roles in immune suppression and immune cell exclusion within the tumor microenvironment [45,46], and include macrophages and DC, T, NK, and myeloid-derived suppressor cells [47]. In a mouse model of HCC, β-catenin pathway activation promotes immune escape and resistance to anti-PD-1 treatment through Wnt/β-catenin signaling; this resulted in defective recruitment of DCs and impaired T cell activity, in turn leading to an impaired anti-tumor immune response [48]. The Wnt and Hippo pathways play important role in HCC development [20,49,50,51]; our results confirm these findings and suggest that more aggressive and specific treatment options are needed for the poor survival group. 

In HCC, *TP53* loss of function is associated with high levels of centrosome amplification, aneuploidy cell proliferation, and chromosome instability [10,52], as well as poor prognosis [53]. In the current study, the HCCW3 subclass had lower *TP53* mutation frequencies; patients in this group had a better prognosis than those in the other groups. Studies assessing the prognostic impact of *TP53* mutations on the outcomes of gastroesophageal adenocarcinomas have demonstrated conflicting results [54,55,56]. From these results, we suggest that *TP53* mutations may not completely correlate with poor survival, and other genetic alterations may also play a role for the survival of patient as our two patients with *TP53* mutations in the HCCW3 group with a good prognosis.

In this study, we obtained a list of Wnt–Hippo pathways-related genes which may influence the infiltrations of immune cells between the good and poor prognosis groups. We also found higher proportion of Th1, NKT, and class-switched memory B-cells in the poor prognosis group. NKT cells contribute to fibrosis progression in nonalcoholic fatty liver disease [57]. A total of 110 upregulated Wnt–Hippo signaling pathway-related genes were identified in our poor prognosis group. Among these genes, the 10 most significant node genes were *WNT8B*, *SMARCA4*, *ARID1A*, *SMARCB1*, *SMARCC2*, *SMARCD1*, *SMARCD2*, *SMARCD3*, *FZD2*, and *WNT9A*. These genes may play important regulatory roles in immune cell migration, differentiation, and activation in HCC. Furthermore, we also conducted RNA-Seq in 12 normal samples. The expression of 88 genes were significantly higher in HCC tissues than that in adjacent nontumor tissues (82 genes in Wnt signaling pathway and 6 genes in Hippo signaling pathway), while the expression of 40 genes were significantly lower in HCC tissues than that in adjacent nontumor tissues (37 genes in Wnt signaling pathway and 3 genes in Hippo signaling pathway) (Supplementary Table S4). These alterations may be used as markers for the diagnosis of HCC and need more cases to confirm it.

## 5. Conclusions

In summary, comprehensive and integrated approaches, such as that developed in this study, will lead to a better understanding of the molecular mechanisms of HCC, and could in turn lead to the discovery of new therapeutic strategies. The results of this study highlight the uniqueness of Taiwanese HCC patients; this uniqueness arises from complex interactions among genetic, lifestyle, and environmental conditions that have arisen in the past 40 years. 

## Figures and Tables

**Table 1:** Association between molecular subgroups HCCW1–4 and clinical variables.

Variable		HCCW1	HCCW2	HCCW3	HCCW4	*p*-Value
Edmondson–Steiner grade	I, II	13	28	21	8	0.0007
	III, IV	5	2	13	10	0.0203
Pathologic stage	I, II	16	27	31	15	>0.05
	III, IV	2	3	3	3	
Macrovascular invasion	No	16	22	24	12	>0.05
	Yes	2	8	9	3	
Cirrhosis	No	3	0	2	2	>0.05
	Yes	15	30	32	16	
Fatty liver	No	3	0	2	3	>0.05
	Yes	15	30	32	15	
HBV	No	7	18	13	10	>0.05
	Yes	11	12	21	8	
HCV	No	12	17	21	10	>0.05
	Yes	6	13	13	8	
AFP	Normal	5	8	7	3	>0.05
	Abnormal	13	22	27	15	
*CTNNB1*	Wild-type	16	29	26	15	0.0586
	Mutation	2	1	8	3	
*TERT*	Wild-type	11	11	20	9	>0.05
	Mutation	7	19	14	9	
*TP53*	Wild-type	12	21	32	13	0.0049
	Mutation	6	9	2	5	
Hoshida	S3	18	11	25	5	0.0526
	S1, S2	0	19	9	13	0.0039
Bidkhor	iHCC1, 2	30	30	33	12	<0.0001
	iHCC3	0	0	1	6	

*p*-value by Fisher’s exact test.

## Data Availability

The RNA-Seq data from this study was submitted to the NCBI Sequence Read Archive (SRA) under BioProject accession nos. PRJNA866195.

## Supplementary Materials

The following supporting information can be downloaded at: https://www.mdpi.com/article/10.3390/cancers14194580/s1, Table S1: Details descriptions of the 272 genes correlated with molecular classification and patient’s survival among 100 Taiwanese HCCs and TCGA-LIHC. Significant genes are those with *p*-value < 0.05; Table S2: Differential expression of Wnt–Hippo pathway-related genes between the HCCW4 (poorest prognosis) and HCCW1-3 subgroups. Significant genes are those with *p*-value < 0.05; Table S3: Differential expression of Wnt–Hippo pathway-related genes between the HCCW3 (best prognosis) and HCCW1, 2, 4 subgroups. Significant genes are those with *p*-value < 0.05; Table S4: The expression difference of Wnt–Hippo signaling pathway-related genes between HCC and normal tissues; Figure S1: Expression of Wnt signaling pathway-related genes in CTNNB1 overexpression and down-expression. *: *p* < 0.05, **: *p* < 0.005, ***: *p* < 0.0005; Figure S2: Expression of Hippo signaling pathway-related genes in YAP1 overexpression and down-expression. *: *p* < 0.05, **: *p* < 0.005, ***: *p* < 0.0005.

## References

[1] Sung H., Ferlay J., Siegel R.L., Laversanne M., Soerjomataram I., Jemal A., Bray F. (2021). Global Cancer Statistics 2020: GLOBOCAN Estimates of Incidence and Mortality Worldwide for 36 Cancers in 185 Countries. CA Cancer J. Clin..

[2] Siegel R.L., Miller K.D., Fuchs H.E., Jemal A. (2021). Cancer Statistics, 2021. CA Cancer J. Clin..

[3] McGlynn K.A., Petrick J.L., El-Serag H.B. (2021). Epidemiology of Hepatocellular Carcinoma. Hepatology.

[4] Llovet J.M., Kelley R.K., Villanueva A., Singal A.G., Pikarsky E., Roayaie S., Lencioni R., Koike K., Zucman-Rossi J., Finn R.S. (2021). Hepatocellular carcinoma. Nat. Rev. Dis. Primers.

[5] Yu M.W., Lin C.L., Liu C.J., Yang S.H., Tseng Y.L., Wu C.F. (2017). Influence of Metabolic Risk Factors on Risk of Hepatocellular Carcinoma and Liver-Related Death in Men with Chronic Hepatitis B: A Large Cohort Study. Gastroenterology.

[6] Anstee Q.M., Reeves H.L., Kotsiliti E., Govaere O., Heikenwalder M. (2019). From NASH to HCC: Current concepts and future challenges. Nat. Rev. Gastroenterol. Hepatol..

[7] Muller M., Bird T.G., Nault J.C. (2020). The landscape of gene mutations in cirrhosis and hepatocellular carcinoma. J. Hepatol..

[8] Friedman S.L., Neuschwander-Tetri B.A., Rinella M., Sanyal A.J. (2018). Mechanisms of NAFLD development and therapeutic strategies. Nat. Med..

[9] Letouze E., Shinde J., Renault V., Couchy G., Blanc J.F., Tubacher E., Bayard Q., Bacq D., Meyer V., Semhoun J. (2017). Mutational signatures reveal the dynamic interplay of risk factors and cellular processes during liver tumorigenesis. Nat. Commun..

[10] Cancer Genome Atlas Research Network (2017). Comprehensive and Integrative Genomic Characterization of Hepatocellular Carcinoma. Cell.

[11] Candia J., Bayarsaikhan E., Tandon M., Budhu A., Forgues M., Tovuu L.O., Tudev U., Lack J., Chao A., Chinburen J. (2020). The genomic landscape of Mongolian hepatocellular carcinoma. Nat. Commun..

[12] Xue R., Chen L., Zhang C., Fujita M., Li R., Yan S.M., Ong C.K., Liao X., Gao Q., Sasagawa S. (2019). Genomic and Transcriptomic Profiling of Combined Hepatocellular and Intrahepatic Cholangiocarcinoma Reveals Distinct Molecular Subtypes. Cancer Cell.

[13] Chaisaingmongkol J., Budhu A., Dang H., Rabibhadana S., Pupacdi B., Kwon S.M., Forgues M., Pomyen Y., Bhudhisawasdi V., Lertprasertsuke N. (2017). Common Molecular Subtypes Among Asian Hepatocellular Carcinoma and Cholangiocarcinoma. Cancer Cell.

[14] Lin C.W., Lin C.C., Mo L.R., Chang C.Y., Perng D.S., Hsu C.C., Lo G.H., Chen Y.S., Yen Y.C., Hu J.T. (2013). Heavy alcohol consumption increases the incidence of hepatocellular carcinoma in hepatitis B virus-related cirrhosis. J. Hepatol..

[15] Chang M.H., You S.L., Chen C.J., Liu C.J., Lai M.W., Wu T.C., Wu S.F., Lee C.M., Yang S.S., Chu H.C. (2016). Long-term Effects of Hepatitis B Immunization of Infants in Preventing Liver Cancer. Gastroenterology.

[16] Liao S.H., Chen C.L., Hsu C.Y., Chien K.L., Kao J.H., Chen P.J., Chen T.H., Chen C.H. (2021). Long-term effectiveness of population-wide multifaceted interventions for hepatocellular carcinoma in Taiwan. J. Hepatol..

[17] Chen C.J., Yang Y.H., Lin M.H., Lee C.P., Tsan Y.T., Lai M.N., Yang H.Y., Ho W.C., Chen P.C., Health Data Analysis in Taiwan Research Group (2018). Herbal medicine containing aristolochic acid and the risk of hepatocellular carcinoma in patients with hepatitis B virus infection. Int. J. Cancer.

[18] Seyda Seydel G., Kucukoglu O., Altinbasv A., Demir O.O., Yilmaz S., Akkiz H., Otan E., Sowa J.P., Canbay A. (2016). Economic growth leads to increase of obesity and associated hepatocellular carcinoma in developing countries. Ann. Hepatol..

[19] Huang S.F., Chang I.C., Hong C.C., Yen T.C., Chen C.L., Wu C.C., Tsai C.C., Ho M.C., Lee W.C., Yu H.C. (2018). Metabolic risk factors are associated with non-hepatitis B non-hepatitis C hepatocellular carcinoma in Taiwan, an endemic area of chronic hepatitis B. Hepatol. Commun..

[20] He S., Tang S. (2020). WNT/beta-catenin signaling in the development of liver cancers. Biomed. Pharmacother..

[21] Liu Y., Wang X., Yang Y. (2020). Hepatic Hippo signaling inhibits development of hepatocellular carcinoma. Clin. Mol. Hepatol..

[22] Sohn B.H., Shim J.J., Kim S.B., Jang K.Y., Kim S.M., Kim J.H., Hwang J.E., Jang H.J., Lee H.S., Kim S.C. (2016). Inactivation of Hippo Pathway Is Significantly Associated with Poor Prognosis in Hepatocellular Carcinoma. Clin. Cancer Res..

[23] Moeini A., Torrecilla S., Tovar V., Montironi C., Andreu-Oller C., Peix J., Higuera M., Pfister D., Ramadori P., Pinyol R. (2019). An Immune Gene Expression Signature Associated with Development of Human Hepatocellular Carcinoma Identifies Mice That Respond to Chemopreventive Agents. Gastroenterology.

[24] Bidkhori G., Benfeitas R., Klevstig M., Zhang C., Nielsen J., Uhlen M., Boren J., Mardinoglu A. (2018). Metabolic network-based stratification of hepatocellular carcinoma reveals three distinct tumor subtypes. Proc. Natl. Acad. Sci. USA.

[25] Hoshida Y., Nijman S.M., Kobayashi M., Chan J.A., Brunet J.P., Chiang D.Y., Villanueva A., Newell P., Ikeda K., Hashimoto M. (2009). Integrative transcriptome analysis reveals common molecular subclasses of human hepatocellular carcinoma. Cancer Res..

[26] Llovet J.M., Montal R., Sia D., Finn R.S. (2018). Molecular therapies and precision medicine for hepatocellular carcinoma. Nat. Rev. Clin. Oncol..

[27] Chang Y.S., Tu S.J., Yen J.C., Lee Y.T., Fang H.Y., Chang J.G. (2021). The Fusion Gene Landscape in Taiwanese Patients with Non-Small Cell Lung Cancer. Cancers.

[28] Chang Y.S., Tu S.J., Chiang H.S., Yen J.C., Lee Y.T., Fang H.Y., Chang J.G. (2020). Genome-Wide Analysis of Prognostic Alternative Splicing Signature and Splicing Factors in Lung Adenocarcinoma. Genes.

[29] Bolger A.M., Lohse M., Usadel B. (2014). Trimmomatic: A flexible trimmer for Illumina sequence data. Bioinformatics.

[30] Kim D., Paggi J.M., Park C., Bennett C., Salzberg S.L. (2019). Graph-based genome alignment and genotyping with HISAT2 and HISAT-genotype. Nat. Biotechnol..

[31] Kovaka S., Zimin A.V., Pertea G.M., Razaghi R., Salzberg S.L., Pertea M. (2019). Transcriptome assembly from long-read RNA-seq alignments with StringTie2. Genome Biol..

[32] Rouillard A.D., Gundersen G.W., Fernandez N.F., Wang Z., Monteiro C.D., McDermott M.G., Ma’ayan A. (2016). The harmonizome: A collection of processed datasets gathered to serve and mine knowledge about genes and proteins. Database.

[33] Gu C., Chen J., Dang X., Chen C., Huang Z., Shen W., Shi X., Dai C., Chen C. (2021). Hippo Pathway Core Genes Based Prognostic Signature and Immune Infiltration Patterns in Lung Squamous Cell Carcinoma. Front. Oncol..

[34] Chin C.H., Chen S.H., Wu H.H., Ho C.W., Ko M.T., Lin C.Y. (2014). cytoHubba: Identifying hub objects and sub-networks from complex interactome. BMC Syst. Biol..

[35] Shannon P., Markiel A., Ozier O., Baliga N.S., Wang J.T., Ramage D., Amin N., Schwikowski B., Ideker T. (2003). Cytoscape: A software environment for integrated models of biomolecular interaction networks. Genome Res..

[36] Aran D., Hu Z., Butte A.J. (2017). xCell: Digitally portraying the tissue cellular heterogeneity landscape. Genome Biol..

[37] Pancho A., Aerts T., Mitsogiannis M.D., Seuntjens E. (2020). Protocadherins at the Crossroad of Signaling Pathways. Front. Mol. Neurosci..

[38] Walle P., Mannisto V., de Mello V.D., Vaittinen M., Perfilyev A., Hanhineva K., Ling C., Pihlajamaki J. (2019). Liver DNA methylation of FADS2 associates with FADS2 genotype. Clin. Epigenet..

[39] Ying J., Li H., Seng T.J., Langford C., Srivastava G., Tsao S.W., Putti T., Murray P., Chan A.T., Tao Q. (2006). Functional epigenetics identifies a protocadherin PCDH10 as a candidate tumor suppressor for nasopharyngeal, esophageal and multiple other carcinomas with frequent methylation. Oncogene.

[40] Fang S., Huang S.F., Cao J., Wen Y.A., Zhang L.P., Ren G.S. (2013). Silencing of PCDH10 in hepatocellular carcinoma via de novo DNA methylation independent of HBV infection or HBX expression. Clin. Exp. Med..

[41] Zhang T., Guan G., Chen T., Jin J., Zhang L., Yao M., Qi X., Zou J., Chen J., Lu F. (2018). Methylation of PCDH19 predicts poor prognosis of hepatocellular carcinoma. Asia Pac. J. Clin. Oncol..

[42] Dang Z., Shangguan J., Zhang C., Hu P., Ren Y., Lv Z., Xiang H., Wang X. (2016). Loss of protocadherin-17 (PCDH-17) promotes metastasis and invasion through hyperactivation of EGFR/MEK/ERK signaling pathway in hepatocellular carcinoma. Tumor Biol..

[43] Lv J., Zhu P., Yang Z., Li M., Zhang X., Cheng J., Chen X., Lu F. (2015). PCDH20 functions as a tumour-suppressor gene through antagonizing the Wnt/beta-catenin signalling pathway in hepatocellular carcinoma. J. Viral Hepat..

[44] Uhlen M., Zhang C., Lee S., Sjostedt E., Fagerberg L., Bidkhori G., Benfeitas R., Arif M., Liu Z., Edfors F. (2017). A pathology atlas of the human cancer transcriptome. Science.

[45] Goldsberry W.N., Londono A., Randall T.D., Norian L.A., Arend R.C. (2019). A Review of the Role of Wnt in Cancer Immunomodulation. Cancers.

[46] Patel S., Alam A., Pant R., Chattopadhyay S. (2019). Wnt Signaling and Its Significance within the Tumor Microenvironment: Novel Therapeutic Insights. Front. Immunol..

[47] Suryawanshi A., Hussein M.S., Prasad P.D., Manicassamy S. (2020). Wnt Signaling Cascade in Dendritic Cells and Regulation of Anti-tumor Immunity. Front. Immunol..

[48] Ruiz de Galarreta M., Bresnahan E., Molina-Sanchez P., Lindblad K.E., Maier B., Sia D., Puigvehi M., Miguela V., Casanova-Acebes M., Dhainaut M. (2019). beta-Catenin Activation Promotes Immune Escape and Resistance to Anti-PD-1 Therapy in Hepatocellular Carcinoma. Cancer Discov..

[49] Perugorria M.J., Olaizola P., Labiano I., Esparza-Baquer A., Marzioni M., Marin J.J.G., Bujanda L., Banales J.M. (2019). Wnt-beta-catenin signalling in liver development, health and disease. Nat. Rev. Gastroenterol. Hepatol..

[50] Kim W., Khan S.K., Gvozdenovic-Jeremic J., Kim Y., Dahlman J., Kim H., Park O., Ishitani T., Jho E.H., Gao B. (2017). Hippo signaling interactions with Wnt/beta-catenin and Notch signaling repress liver tumorigenesis. J. Clin. Investig..

[51] Manmadhan S., Ehmer U. (2019). Hippo Signaling in the Liver—A Long and Ever-Expanding Story. Front. Cell Dev. Biol..

[52] Rao C.V., Asch A.S., Yamada H.Y. (2017). Frequently mutated genes/pathways and genomic instability as prevention targets in liver cancer. Carcinogenesis.

[53] Liu J., Ma Q., Zhang M., Wang X., Zhang D., Li W., Wang F., Wu E. (2012). Alterations of TP53 are associated with a poor outcome for patients with hepatocellular carcinoma: Evidence from a systematic review and meta-analysis. Eur. J. Cancer.

[54] Lim B.H., Soong R., Grieu F., Robbins P.D., House A.K., Iacopetta B.J. (1996). p53 accumulation and mutation are prognostic indicators of poor survival in human gastric carcinoma. Int. J. Cancer.

[55] Blanchet A., Bourgmayer A., Kurtz J.E., Mellitzer G., Gaiddon C. (2021). Isoforms of the p53 Family and Gastric Cancer: A Menage a Trois for an Unfinished Affair. Cancers.

[56] Deng W., Hao Q., Vadgama J., Wu Y. (2021). Wild-Type TP53 Predicts Poor Prognosis in Patients with Gastric Cancer. J. Cancer Sci. Clin Ther.

[57] Syn W.K., Oo Y.H., Pereira T.A., Karaca G.F., Jung Y., Omenetti A., Witek R.P., Choi S.S., Guy C.D., Fearing C.M. (2010). Accumulation of natural killer T cells in progressive nonalcoholic fatty liver disease. Hepatology.

